# Epidemiology, risk factors, and co-infection of vector-borne pathogens in goats from Sistan and Baluchestan province, Iran

**DOI:** 10.1371/journal.pone.0218609

**Published:** 2019-06-20

**Authors:** Hassan Hakimi, Ali Sarani, Mika Takeda, Osamu Kaneko, Masahito Asada

**Affiliations:** 1 Department of Protozoology, Institute of Tropical Medicine (NEKKEN), Nagasaki University, Nagasaki, Japan; 2 Department of Clinical Science, University of Zabol, Veterinary Faculty, Zabol, Iran; University of Minnesota, UNITED STATES

## Abstract

Several vector-borne pathogens restrict livestock farming and have significant economic impact worldwide. In endemic areas livestock are exposed to different tick species carrying various pathogens which could result in co-infection with several tick-borne pathogens in a single host. Although the co-infection of and the interaction among pathogens are critical factors to determine the disease outcome, pathogen interactions in the vector and the host are poorly understood. In this study, we surveyed the presence of *Babesia ovis*, *Theileria ovis*, *Theileria lestoquardi*, *Anaplasma ovis*, *Anaplasma phagocytophilum*, and *Anaplasma marginale* in 200 goats from 3 different districts in Sistan and Baluchestan province, Iran. Species-specific diagnostic PCRs and sequence analysis revealed that 1.5%, 12.5%, and 80% of samples were positive for *T*. *lestoquardi*, *T*. *ovis*, and *A*. *ovis*, respectively. Co-infections of goats with up to 3 pathogens were seen in 22% of the samples. We detected a significant association between *T*. *ovis* infection and age, *T*. *ovis* infection and location (Zabol), and *A*. *ovis* infection and location (Sarbaz) by multivariate logistic regression analysis. In addition, by analyzing the data with respect to *Plasmodium caprae* infection in these goats, a negative correlation was found between *P*. *caprae* and *A*. *ovis* infection. This study contributes to understanding the epidemiology of vector-borne pathogens and their interplay in goats.

## Introduction

Tick-borne diseases remain an economic burden for the livestock industry of tropical and subtropical regions of the world. Protozoan parasites such as *Babesia* spp. and *Theileria* spp. together with *Anaplasma* spp. are responsible for tick-borne diseases in small ruminants and cause great economic losses in the livestock and livestock-related industries [1, 2].

Small ruminant theileriosis is mainly caused by *Theileria lestoquardi*, *Theileria ovis*, and *Theileria separata*. *T*. *lestoquardi* is the most virulent specie and occasionally causes death while *T*. *ovis* and *T*. *separata* are benign and cause subclinical infections in small ruminants [3]. Several species of *Babesia* have been described to cause ovine and caprine babesiosis including *Babesia ovis*, *Babesia motasi*, *Babesia crassa*, and *Babesia foliata* [4]. *B*. *ovis* is the most pathogenic and causes fever, hemoglobinuria, severe anemia, icterus and occasional death [5]. *Anaplasma* spp. are important for human and animal health and these pathogens are generally considered to produce mild clinical symptoms. Although several *Anaplasma* spp. including *Anaplasma marginale*, *Anaplasma ovis*, and *Anaplasma phagocytophilum* could be found in small ruminants, *A*. *ovis* is the main cause of small ruminant anaplasmosis in the world.

In Iran small ruminant farming is widely practiced with 52 and 26 million heads of sheep and goats, respectively, being raised mainly by small-scale farmers [6]. In regions with harsh and severe environments, such as central and southeast Iran, goat raising dominates. The great diversity of the environment in Iran affects the distribution of ticks, and thereby the pathogens transmitted. Several epidemiological studies are available regarding tick-borne pathogens in small ruminants in Northern and Western regions in Iran [7–10]. However, there is a scarcity of data regarding the prevalence of *Babesia*, *Theileria*, and *Anaplasma* spp. infecting small ruminants in southeastern Iran. This study investigated the prevalence of tick-borne pathogens in Sistan and Baluchestan province, in the southeastern part of Iran where it borders with Afghanistan and Pakistan; and where the frequent border-crossing animal passage facilitates the circulation of tick-borne pathogens between countries [11].

In endemic areas livestock are bitten by vectors carrying multiple pathogens or different vectors transmitting various pathogens, which result in co-infections in the host. The interaction among different pathogens within a host are complex and may result in protection against virulent pathogens or exacerbate the clinical symptoms [12]. In a study done on the indigenous African cattle in Kenya, it was shown that co-infection with less pathogenic *Theileria* spp. in calves results in a decreased mortality associated with virulent *T*. *parva* which is likely the result of cross protection [13]. A recent study also showed a negative interaction between *B*. *ovis* and *T*. *ovis* in sheep, indicating infection with the less pathogenic *T*. *ovis* produces protection against highly pathogenic *B*. *ovis* [14]. In contrast, co-infection of *B*. *ovata* and *T*. *orientalis*, two parasites which are transmitted via the same tick species, exacerbate the symptoms and produce clinical anemia in cattle [15]. Recently we identified the goat malaria parasite, *Plasmodium caprae*, in goat samples originating from Sistan and Baluchestan province, Iran [16]. However, nothing is known for this pathogen except some DNA sequence and morphology. In order to gain insights into pathogenicity and interaction of this parasite with other vector-borne pathogens in the host, we examined the prevalence of tick-borne piroplasms and *Anaplasma* spp. in these goat samples and evaluated the interplay among the identified pathogens.

## Materials and methods

### Sampling sites and blood collection

Blood samples were collected from 200 goats (95 males and 105 females) from 3 districts in Sistan and Baluchestan province, including Zabol (n = 51), Sarbaz (n = 125), and Chabahar (n = 24) as shown in S1 Fig. These samples were utilized for screening of *Plasmodium caprae* in this region [16]. Age of the goats were recorded by 0.5-year interval based on the owner’ report. Average ages were 1.9 years old (range: 1–5 years old) for male and 3.0 years old (range: 1–6 years old) for female goats. In each district, blood samples were collected from different farms. Sampling was done in January, June, and November of 2016 and July of 2017. Blood sampling and DNA extraction was performed as described [16].

### Ethical statement

Sampling of goats was performed with the informed consent of the farm owners. All procedures were carried out in compliance to the ethical guidelines for the usage of animal samples of University of Zabol. This study was approved by the Ethics Committee of University of Zabol (permission number: IRUOZ.ECRA.2016.01).

### Detection of *Babesia* spp. *Theileria* spp. and *Anaplasma* spp. by species-specific PCR

Each DNA sample was screened for *B*. *ovis*, *T*. *lestoquardi*, *T*. *ovis*, *A*. *phagocytophilum*, *A*. *ovis*, and *A*. *marginale* by species-specific PCR as described [17–21]. Small subunit ribosomal RNA (SSUrRNA) was the target gene for detection of *B*. *ovis* and *T*. *ovis* while specific primers targeting merozoite surface antigen gene (*ms1*) were used for detection of *T*. *lestoquardi*. *A*. *ovis*, and *A*. *marginale* were screened using primers targeting major surface protein 4 gene (*msp-4*); and for specific detection of *A*. *phagocytophilum*, primers targeting *epank1* gene were used (Table 1).

**Table 1:** List of primers used in this study.

Target	Primer sequences		Fragment (bp)	Anealing temp (°C)	Reference
	Forward	Reverse			
*B*. *ovis* SSUrRNA	TGGGCAGGACCTTGGTTCTTCT	CCGCGTAGCGCCGGCTAAATA	549	62	Aktas et al. (2005) [17]
*T*. *ovis* SSUrRNA	TCGAGACCTTCGGGT	TCCGGACATTGTAAAACAAA	520	60	Aktas et al. (2006) [19]
*T*. *lestoquardi* ms1-2	GTGCCGCAAGTGAGTCA	GGACTGATGAGAAGACGATGAG	730	55	Taha et al. (2011) [18]
*A*. *ovis* msp-4	TGAAGGGAGCGGGGTCATGGG	GAGTAATTGCAGCCAGGCACTCT	347	62	Torina et al. (2012) [21]
*A*. *phagocytophilum* epank1	GAGAGATGCTTATGGTAAGAC	CGTTCAGCCATCATTGTGAC	444	54–62 (Touch-down PCR)	Walls et al. (2000) [20]
*A*. *marginale* msp-4	CTGAAGGGGGAGTAATGGG	GGTAATAGCTGCCAGAGATTCC	344	60	Torina et al. (2012) [21]

### Cloning and sequencing

PCR products of all positive samples for *T*. *ovis* or *T*. *lestoquardi* and three positive samples for *A*. *ovis* randomly selected from each sampling village (21 samples in total) were sequenced. The amplified PCR products were recovered from agarose gels and cloned into the Zero Blunt TOPO vector (Thermo Fisher Scientific, Carlsbad, CA, USA) according to the manufacturer’s protocol. Following transformation three *E*. *coli* colonies were selected, the plasmids were extracted and purified, and the gene sequences were analyzed using BigDye Terminator v1.1 and an ABI 3730 DNA Analyzer (Applied Biosystems, CA, USA). The single nucleotide polymorphisms (SNPs) found in the obtained sequence were confirmed by repeating the amplification, cloning, and sequencing process. *T*. *lestoquardi ms1* (*Tlms1*), *T*. *ovis* SSUrRNA (*To*SSUrRNA), and *A*. *ovis msp-4* (*Aomsp-4*) sequences from this study were deposited in GenBank (*T*. *ovis*: LC430938 and LC430939, *A*. *ovis*: LC430940, LC430941 and LC430942, *T*. *lestoquardi*: LC430943, LC430944, LC430945, LC430946, LC430947 and LC430948).

### Statistical analysis

The associations of pathogens with sex, age, and sampling location were analyzed by two-tailed Fisher’s exact test and logistic regression analysis. Factors with at least borderline significance (p < 0.15) according to univariate analysis were included in the multivariate analysis. Backward-stepwise elimination was used to generate a minimum adequate model and excluded variables (p > 0.05) were retested in the minimum model. Two-tailed Fisher’s exact test and logistic regression analysis were also used to evaluate the significance of association between co-infected pathogens. A correlation coefficient (Rij) between the different pairs of pathogens was measured as described [22]. Twenty-seven negative goats were excluded from the calculation of correlation coefficient. Statistical analysis was performed using EZR version 1.27 [23].

## Results and discussion

### Prevalence of *T*. *lestoquardi*, *T*. *ovis*, *B*. *ovis* and *Anaplasma* spp. in goat blood samples from Sistan and Baluchestan province, Iran

All 200 samples analyzed were negative for *B*. *ovis*, *A*. *marginale*, and *A*. *phagocytophilum*; while 3 (1.5%), 25 (12.5%), and 160 (80%) were positive for *T*. *lestoquardi*, *T*. *ovis*, and *A*. *ovis*, respectively (Table 2). In Zabol, 19/51 (37.3%) and 50/51 (98%) samples were positive for *T*. *ovis* and *A*. *ovis*, respectively, while *T*. *lestoquardi* was not detected. In Sarbaz, 3/125 (2.4%), 6/125 (4.8%) and 87/125 (69.6%) samples were positive for *T*. *lestoquardi*, *T*. *ovis* and *A*. *ovis*, respectively. None of the samples from Chabahar were positive for *T*. *lestoquardi* and *T*. *ovis* while 23/24 (95.8%) were positive for *A*. *ovis*. Sequence analysis of obtained PCR products confirmed that the species identities judged by PCR diagnosis were correct. In spite of low infection rate of *T*. *lestoquardi*, 6 different nucleotide sequences of *ms1* was obtained which were relatively diversified and showed 97.9–99.7% identity values. Two nucleotide sequences for ToSSUrRNA and 3 nucleotide sequences for 3 *Aomsp-4* identified in this study were relatively conserved with 99.8% and 99.3–99.7% sequence identity, respectively.

**Table 2:** Pathogens identified in different districts in Sistan and Baluchestan province.

District	Pathogens			
	*T*. *ovis*	*T*. *lestoquardi*	*A*. *ovis*	Total samples
Zabol	19 (37.3%)	0	50 (98%)	51
Sarbaz	6 (4.8%)	3 (2.4%)	87 (69.6%)	125
Chabahar	0	0	23 (95.8%)	24
Total	25 (12.5%)	3 (1.5%)	160 (80%)	200

Several species of *Theileria* can infect small ruminants and *T*. *ovis* and *T*. *lestoquardi* were reported previously from Iran [8, 24, 25]. While there is no report on *T*. *ovis* prevalence in the goat population in Iran, the prevalence of *T*. *lestoquardi* is 6.25% (by semi-nested PCR diagnosis targeting SSUrRNA) and 19% (by microscopic diagnosis with Giemsa-stained smear) in West Azerbaijan and Kurdistan provinces, respectively, in western Iran [26, 27]. The prevalence of *T*. *lestoquardi* in sheep ranges from 6.6% in Razavi Khorasan province in northeast Iran to 33% in Fars province in central Iran, which is one of the most important endemic regions for ovine theileriosis in Iran [8, 28]. *T*. *ovis* is more prevalent in sheep and ranges from 13.2% in western Iran to 73% in central Iran [8, 28]. The overall infection rate of *T*. *lestoquardi* in this study was 1.5%, which is relatively low compared to the previous reports from Iran [26, 27]. This difference may originate from the difference in sampling time, as all the positive samples in this study were collected in summer. The climate diversity which affects the distribution and infestation of the tick vector in various regions in Iran [29] may also contribute to a difference in infection rates. Detection method could be another possible contributing factor to this difference. *T*. *ovis* was present in 12.5% of samples and this is the first molecular report of this parasite in goats in Iran. *B*. *ovis* is one of the most important and highly pathogenic parasites that infects small ruminants and prevalent in different regions in Iran [9]. Although *Rhipicephalus bursa*, the tick vector of *B*. *ovis*, exists in Sistan and Baluchestan [29], we could not detect this pathogen in this study suggesting that *B*. *ovis* may not be common in the surveyed region.

Goat anaplasmosis in Iran is mainly caused by *A*. *ovis* and *A*. *marginale* [10]. The infection rate of *A*. *ovis* is from 34.7% (by nested PCR diagnosis targeting *msp4*) in western Iran to 63.7% (by PCR-RFLP analysis targeting *msp4*) in northern and northeastern Iran [10, 30]. The overall infection rate of *A*. *ovis* in goats was 80% in this study which was higher than other reported areas in Iran. The difference in sampling time, diagnosis method, geographical, and climate variation may contribute to the differential prevalence of this pathogen.

A higher prevalence of *T*. *ovis* infection in goats with >1 year of age was observed (one positive out of 39 goats ≤1 years old, 24 positive out of 161 goats >1 years old were positive, p < 0.05 by two-tailed Fisher’s exact test), however no statistical differences were observed between the prevalence of other pathogens and the age of goats (Table 3). In a study from goats in Saudi Arabia, *T*. *ovis* was significantly less prevalent in animals <1 year of age [31]. Similarly, *T*. *ovis* infection was more prevalent in the goats > 1 year old in Turkey [32]. The higher prevalence of *T*. *ovis* in adults (> 1 year) may be due to a more frequent contact of adult goats to the tick vector. Additionally, male goats were significantly more frequently infected with *T*. *ovis* (17 out of 95 male goats and 8 out of 105 female goats were positive) and *A*. *ovis* (87 out of 95 male goats and 73 out of 105 female goats were positive) (p < 0.05 by two-tailed Fisher’s exact test; Table 3), which is consistent to the previous report for *T*. *ovis* [31] and *A*. *ovis* [32]. Differences in the management of male and female animals during pregnancy, labor, and lactation may affect the degree of the exposure to tick vectors, which in turn may contribute to higher prevalence of these pathogens in male animals [33]. The prevalence of pathogen among the sampling locations were different for *T*. *ovis* and *A*. *ovis* (p < 0.05 by two-tailed Fisher’s exact test). These correlations were further analyzed by multivariate logistic regression analysis. Significant association between *T*. *ovis* infection and age, *T*. *ovis* infection and Zabol, and *A*. *ovis* infection and Sarbaz was detected by this analysis, whereas significant correlation between pathogen and sex was not detected (Table 4). Zabol is located around 500 km north of Sarbaz and Chabahar with a drier climate which may affect the distribution of ticks and subsequently *T*. *ovis*. However, the data showed that *T*. *ovis* and *A*. *ovis* infections correlate with different locations suggesting other factor such as difference in farm management and tick control situation may also contribute to the prevalence of these pathogens.

**Table 3:** Factors associating with the infection evaluated by Fisher’s exact test.

Pathogens		Age		Odds ratio	95% Confidential interval	p value	Sex		Odds ratio	95% Confidential interval	p value	Location
		≤1	>1				Male	Female				p value
*T*. *ovis*	Positive	1	24	0.15	0.0036–0.99	**0.033**	17	8	2.63	1.01–7.43	**0.033**	**0.0001**
	Negative	38	137				78	97				
*T*. *lestoquardi*	Positive	1	2	2.08	0.035–40.98	0.48	2	1	2.23	0.11–133.1	0.61	0.70
	Negative	38	159				93	104				
*A*. *ovis*	Positive	33	127	1.47	0.55–4.65	0.51	87	73	4.73	1.98–12.65	**0.0001**	**0.0001**
	Negative	6	34				8	32				

**Table 4:** Multivariate logistic regression analysis of the factors associating with the infection.

Pathogens	Factors	Odds ratio	95% Confidential interval	p value
*T*. *ovis*	Age	1.96	2.37–162	0.0057
*T*. *ovis*	Location (Zabol)	2.42	7.91–74	0.0001
*A*. *ovis*	Location (Sarbaz)	0.1	0.013–0.76	0.027

There is no epidemiological report on tick-borne pathogens in small ruminants in Afghanistan, and similar reports are limited from Pakistan and not from the western region [34, 35]. Thus, the result of this study serves as a useful reference to estimate the prevalence of tick-born piroplasms and *Anaplasma* spp. in the Afghanistan and the Pakistan regions neighboring the Sistan and Baluchestan province of Iran.

### Interplay between *P*. *caprae* and other pathogens co-infected in the goat

Because the goat malaria parasite, *P*. *caprae*, was detected in 28 samples among these 200 samples in the previous study [16], we evaluated the possible effect of a specific pathogen infection against the other pathogens identified in this study. A correlation coefficient value (R_ij_) was calculated for each two-pathogen interaction [22]. Co-infections are summarized in Table 5. A strong negative correlation between *P*. *caprae* and *A*. *ovis* infections with R_ij_ value of –0.593 was observed (p < 0.01 by two-tailed Fisher’s exact test, Table 6 and their double infection was only 8%. Infection of *P*. *caprae* and *T*. *ovis* showed a relatively weak negative correlation, yet significant (R_ij_ value: ‒0.182, p < 0.05 by two-tailed Fisher’s exact test). However, all *T*. *ovis* positive samples were also positive for *A*. *ovis* and a relatively weak, though significant, positive correlation was observed (R_ij_ value: 0.118, p < 0.01 by two-tailed Fisher’s exact test). The co-infections were further analyzed by multivariate logistic regression analysis including age, sex, and location information and a significant correlation was detected between *P*. *caprae* and *A*. *ovis* infections (p < 0.05, odds ratio: 0.26, 95% confidence interval: 0.11–0.61).

**Table 5:** Co-infection of pathogens in goat samples from Sistan and Baluchestan province.

Pathogens	Positive numbers (%)
Single infection	
*T*. *lestoquardi*	1 (0.5)
*A*. *ovis*	118 (59)
*P*. caprae*	12 (6)
Double infection	
*A*. *ovis & T*. *ovis*	24 (12)
*A*. *ovis & T*. *lestoquardi*	1 (0.5)
*A*. *ovis & P*. *caprae*	16 (8)
Triple infection	
*A*. *ovis*, *T*. *ovis & T*. *lestoquardi*	1 (0.5)

*From Kaewthamasorn et al, 2018 [16]

**Table 6:** Fisher’s exact test for the coinfection of two pathogens.

Pathogens	*A*. *ovis*	*T*. *ovis*	*T*. *lestoquardi*
*P*. *caprae*	**-0.593 (0.0036)**	**-0.182 (0.029)**	-0.059 (1)
*A*. *ovis*	―	**0.118 (0.0055)**	-0.131 (0.49)
*T*. *ovis*	―	―	-0.072 (0.33)

Correlation coefficient between two pathogens (Rij) is presented. The p-value is shown in the bracket. p-values were calculated by Fisher’s exact test.

The negative correlation between two vector-borne pathogens could also happen in the host or the vector by competing for resources such as space like available erythrocytes, nutrients, or impact on the immune response. We found a negative correlation between *P*. *caprae* and *A*. *ovis*. Mosquitoes are the likely vector for *P*. *caprae*, while *A*. *ovis* and *T*. *ovis* are transmitted by ticks; thus excluding the possibility of negative interference in the vector and highlighting likely competition in the goat [36, 37]. A negative association was reported between *Theileria annulata*, a protozoan parasite responsible for tropical theileriosis, and *A*. *marginale* [22]. In a study that was done using blood samples from sick sheep, the authors showed that the presence of *T*. *ovis* was negatively correlated with *B*. *ovis*, indicating that infection with low pathogenic *T*. *ovis* protects sheep from infection with highly pathogenic *B*. *ovis* [14]. An absolute exclusion was shown to exist between *T*. *annulata* and *B*. *bovis*, since the authors did not find any co-infection in cattle samples in Algeria [22]. The negative correlation between two pathogens could happen through modification of host immune response such as development of cross-protection immunity. Alternatively, this may be due to a mechanical interference between pathogens since all these pathogens infect host erythrocytes. However, there is no data on the erythrocyte type preference and receptors for these pathogens. Studies on the molecular mechanism of erythrocyte invasion and modification mediated by these pathogens would provide important insights behind these observations; however, such information are scarce, if any. Given the fact that *P*. *caprae* observed in the goats had very low parasitemia, below the microscopy detection limit [16], we consider that the interference by *P*. *caprae* against *A*. *ovis* and *T*. *ovis* is quite unlikely and exclusion may take place through modulating the host immune system or mechanical interference by *A*. *ovis* and *T*. *ovis*.

The best example of positive correlation among two vector-borne pathogens is between *Borrelia burgdorferi*, the causative agent of Lyme disease, and *Babesia microti*, the primary agent of human babesiosis, both of which are transmitted by the tick *Ixodes ricinus* [38]. Co-infection of these two pathogens are common and enhance the transmission and emergence of *B*. *microti* in human population in USA, possibly by lowering the ecological threshold for establishment of *B*. *microti* [12, 39]. Immunosuppression by one pathogen may predispose the host to the second pathogen. This phenomenon could be seen in *B*. *microti* with the parasites *Trypanosoma musculi* and *Trichuris muris* in mice [40, 41]. Moreover, the possibility of coinfection increases if the pathogens are transmitted by the same vector [40]. Both *T*. *ovis* and *A*. *ovis* are transmitted by the same tick, *R*. *sanguineus*, in the region thus positive correlation may be a result of the simultaneous inoculation of these pathogens to the goat by ticks or by enhancing pathogen fitness and transmission by tick vector [37, 42]. In addition, positive correlation of *T*. *ovis* and *A*. *ovis* infection may suggests the absence of cross protection between these pathogens; one eukaryotic protozoan parasite and the other prokaryotic bacteria. One infection appears to increase the susceptibility to the other pathogen. Co-infection is often associated with exacerbation of symptoms, thus, competition among pathogens could be beneficial to the host [15]. It is worth investigating the mechanism for competitive interaction among these pathogens.

## Conclusions

The distribution of *T*. *lestoquardi*, *T*. *ovis*, and *A*. *ovis* in different regions in Iran is well reported. However, in this study we focused in Southeast of Iran, Sistan and Baluchestan province, where no reports exist, and showed the co-infection of these pathogens. Coinfection of several pathogens might influence the pathogenesis in the host and may jeopardize correct diagnoses. We showed a negative correlation between *A*. *ovis* and *P*. *caprae*, suggesting possible interference via immunity or against erythrocyte invasion by the other pathogen. The results of this study may contribute to understand these pathogen interactions in the host, and aid in designing preventive measures of tick-borne pathogens in the region. However, limited sample size is a constraint factor of this study and our findings needs to be extended by studies with large-scale samples from different geographical regions as well as experimental infection studies.

## Supporting information

S1 Fig Map of Iran showing sampling sites in Sistan and Baluchestan province, Iran. (TIF)

## Abbreviations

*ms1*: merozoite surface antigen gene
*msp-4*: major surface protein 4 gene
SNPs: single nucleotide polymorphisms
SSUrRNA: Small subunit ribosomal RNA

## References

[1] Berggoetz M, Schmid M, Ston D, Smith V, Chevillon C, Pretorius AM, et al Tick-borne pathogens in the blood of wild and domestic ungulates in South Africa: Interplay of game and livestock. Ticks Tick-borne Dis. 2014; 5:166–75. 10.1016/j.ttbdis.2013.10.007 24418761

[2] Dantas-Torres F, Chomel BB, Otranto D. Ticks and tick-borne diseases: a One Health perspective. Trends Parasitol. 2012; 28:437–446. 10.1016/j.pt.2012.07.003 22902521

[3] Alessandra T, Santo C. Tick-borne diseases in sheep and goats: clinical and diagnostic aspects. Small Rumin Res. 2012; 106:S6–S11.

[4] Lempereur L, Beck R, Fonseca I, Marques C, Duarte A, Santos M, et al Guidelines for the detection of *Babesia* and *Theileria* parasites. Vector Borne Zoonotic Dis. 2017; 17:51–5. 10.1089/vbz.2016.1955 28055573

[5] Yeruham I, Hadani A, Galker F. Some epizootiological and clinical aspects of ovine babesiosis caused by *Babesia ovis*–a review. Vet Parasitol. 1998; 74:153–163. 9561703 10.1016/s0304-4017(97)00143-x

[6] Valizadeh R. Iranian sheep and goat industry at a glance -in: Karim S.A. & Joshi A. (Eds) Climate changes and stress management: Sheep and goat production. SSPH Pub., India; 2010 p. 431–440.

[7] Razmi GR, Dastjerdi K, Hossieni H, Naghibi A, Barati F, Aslani MR. An epidemiological study on *Anaplasma* infection in cattle, sheep, and goats in Mashhad Suburb Khorasan province, Iran. Ann N Y Acad Sci. 2006; 1078:479–481. 10.1196/annals.1374.089 17114758

[8] Zaeemi M, Haddadzadeh HR, Khazraiinia P, Kazemi B, Bandehpour M. Identification of different *Theileria* species (*Theileria lestoquardi*, *Theileria ovis* and *Theileria annulata*) in naturally infected sheep using nested PCR-RFLP. Parasitol Res. 2011; 108:837–843. 10.1007/s00436-010-2119-0 20978792

[9] Motavalli Haghi M, Etemadifar F, Fakhar M, Teshnizi SH, Soosaraei M, Shokri A, et al Status of babesiosis among domestic herbivores in Iran: A systematic review and meta-analysis. Parasitol Res. 2017; 116:1101–1109. 10.1007/s00436-016-5368-8 28054180

[10] Yousefi A, Rahbari S, Shayan P, Sadeghi-dehkordi Z, Bahonar A. Molecular detection of *Anaplasma marginale* and *Anaplasma ovis* in sheep and goat in west highland pasture of Iran. Asian Pac J Trop Biomed. 2017; 7(5):455–459.

[11] Izadi S, Holakouie-Naieni K, Majdzadeh SR, Chinikar S, Nadim A, Rakhshani F, et al Seroprevalence of Crimean-Congo hemorrhagic fever in Sistan-va-Baluchestan province of Iran. Jpn J Infect Dis. 2006; 59:326–8. 17060701

[12] Diuk-Wasser MA, Vannier E, Krause PJ. Co-infection by *Ixodes* tick-borne pathogens: ecological, epidemiological, and clinical consequences. Trends Parasitol. 2016; 32:30–42. 10.1016/j.pt.2015.09.008 26613664 PMC4713283

[13] Woolhouse MEJ, Thumbi SM, Jennings A, Chase-Topping M, Callaby R, Kiara H, et al Co-infections determine patterns of mortality in a population exposed to parasite infection. Sci Adv. 2015; 1:e1400026–e1400026. 10.1126/sciadv.1400026 26601143 PMC4643819

[14] Sevinc F, Zhou M, Cao S, Ceyla O, Aydin MF, Sevinc M, et al Haemoparasitic agents associated with ovine babesiosis: A possible negative interaction between *Babesia ovis* and *Theileria ovis*. Vet Parasitol. 2018; 252:143–147. 10.1016/j.vetpar.2018.02.013 29559137

[15] Sivakumar T, Tagawa M, Yoshinari T, Ybañez AP, Igarashi I, Ikehara Y, et al PCR Detection of *Babesia ovata* from cattle reared in Japan and clinical significance of coinfection with *Theileria orientalis*. J Clin Microbiol. 2012; 50:2111–3. 10.1128/JCM.00220-12 22442312 PMC3372144

[16] Kaewthamasorn M, Takeda M, Saiwichai T, Gitaka J, Tiawsirisup S, Imasato Y, et al Genetic homogeneity of goat malaria parasites in Asia and Africa suggests their expansion with domestic goat host. Sci Rep. 2018; 8:5827 10.1038/s41598-018-24048-0 29643434 PMC5895593

[17] Aktas M, Altay K, Dumanli N. Development of a polymerase chain reaction method for diagnosis of *Babesia ovis* infection in sheep and goats. Vet Parasitol. 2005; 133:277–281. 10.1016/j.vetpar.2005.05.057 16043298

[18] Taha KM, Salih DA, Ahmed BM, Enan KA, Ali AM, Elhussein AM. First confirmed report of outbreak of malignant ovine theileriosis among goats in Sudan. Parasitol Res. 2011; 109(6):1525–7. 10.1007/s00436-011-2428-y 21537979

[19] Aktas M, Altay K, Dumanli N. PCR-based detection of *Theileria ovis* in *Rhipicephalus bursa* adult ticks. Vet Parasitol. 2006; 140:259–263. 10.1016/j.vetpar.2006.04.005 16682122

[20] Walls JJ, Caturegli P, Bakken JS, Asanovich KM, Dumler JS. Improved sensitivity of PCR for diagnosis of human granulocytic ehrlichiosis using *epank1* genes of *Ehrlichia phagocytophila*-group ehrlichiae. J Clin Microbiol. 2000; 38:354–356. 10618115 10.1128/jcm.38.1.354-356.2000 PMC88723

[21] Torina A, Agnone A, Blanda V, Alongi A, D’Agostino R, Caracappa S, et al Development and validation of two PCR tests for the detection of and differentiation between *Anaplasma ovis* and *Anaplasma marginale*. Ticks Tick Borne Dis. 2012; 3:283–287. 10.1016/j.ttbdis.2012.10.033 23182548

[22] Dib L, Bitam I, Tahri M, Bensouilah M, De Meeus T. Competitive exclusion between piroplasmosis and anaplasmosis agents within cattle. PLoS Pathog. 2008; 4:e7 10.1371/journal.ppat.0040007 18225951 PMC2323288

[23] Kanda Y. Investigation of the freely available easy-to-use software ‘EZR’ for medical statistics. Bone Marrow Transplant. 2013; 48(3):452–8. 10.1038/bmt.2012.244 23208313 PMC3590441

[24] Heidarpour Bami M, Khazraiinia P, Haddadzadeh HR, Kazemi B. Identification of *Theileria* species in sheep in the eastern half of Iran using nested PCR-RFLP and microscopic techniques. Iranian J Vet Res. 2010; 11(3):262–266.

[25] Yaghfoori S, Razmi G, Heidarpour M. Molecular detection of *Theileria* spp. in sheep and vector ticks in Fasa and Kazeroun areas, Fars province, Iran. Arch Razi Instit. 2013; 68:159–164.

[26] Mohammadi SM, Esmaeilnejad B, Jalilzadeh-amin G. Molecular detection, infection rate and vectors of *Theileria lestoquardi* in goats from West Azerbaijan province, Iran. Vet Res Forum. 2017; 8(2):139–144. 28785390 PMC5524552

[27] Hasheminasab SS, Moradi P, Wright I. A four year epidemiological and chemotherapy survey of babesiosis and theileriosis, and tick vectors in sheep, cattle and goats in Dehgolan, Iran. Ann Parasitol. 2018; 64(1):43–48. 29717573 10.17420/ap6401.131

[28] Razmi G, Yaghfoori S. Molecular surveillance of *Theileria ovis*, *Theileria lestoquardi* and *Theileria annulata* infection in sheep and ixodid ticks in Iran. Onderstepoort J Vet. 2013; 80(1):635. 10.4102/ojvr.v80i1.635 24396911

[29] Rahbari S, Nabian S, Shayan P. Primary report on distribution of tick fauna in Iran. Parasitol Res. 2007; 101 (Suppl. 2):175–177. 10.1007/s00436-007-0692-7 17823823

[30] Ahmadi-Hamedani M, Khaki Z, Rahbari S, Kazemi B, Bandehpour M. Molecular identification of anaplasmosis in goats using a new PCR-RFLP method. Iranian J Vet Res. 2009; 10:367–372.

[31] Alanazi AD, Said AE, Ghoneim AM, Alyousif MS, Alanazi IO. A comprehensive evaluation and first molecular report of *Theileria* ovis infection in small ruminants in Saudi Arabia. Trop Anim Health Prod. 2019; 51: 89 10.1007/s11250-018-1663-y 30047010

[32] Zhou M, Cao S, Sevinc F, Sevinc M, Ceylan O, Ekici S, et al Molecular detection and genetic characterization of *Babesia*, *Theileria* and *Anaplasma* amongst apparently healthy sheep and goats in the central region of Turkey. Ticks Tick-Borne Dis. 2017; 8:246–252. 10.1016/j.ttbdis.2016.11.006 27908771

[33] Riaz M, Nazir MM, Tasawar Z, Ahmed AN, Ayaz MM, Akram Q, et al Molecular epidemiology and prevalence of *Theileria lestoquardi* and *Theileria ovis* infection in goats infested with tick vectors from Multan, Pakistan. J Med Entomol 2019; In press. 10.1093/jme/tjy229 30690567

[34] Saeed S, Jahangir M, Fatima M, Shaikh RS, Khattak RM, Ali M, et al PCR based detection of *Theileria lestoquardi* in apparently healthy sheep and goats from two districts in Khyber Pukhtoon Khwa (Pakistan). Trop Biomed. 2015; 32:225–232. 26691250

[35] Iqbal F, Khattak R, Ozubek S, Khattak M, Rasul A, Aktas M. Application of the reverse line blot assay for the molecular detection of *Theileria* and *Babesia* sp. in sheep and goat blood samples from Pakistan. Iran J Parasitol. 2013; 8:289–295. 23914243 PMC3724155

[36] Noaman V. Identification of hard ticks collected from sheep naturally infected with *Anaplasma* ovis in Isfahan province, central Iran. Comp Clin Path. 2012; 21:367–369.

[37] Hosseini-Vasoukolaei N, Oshaghi MA, Shayan P, Vatandoost H, Babamahmoudi F, Yaghoobi-Ershadi MR, et al *Anaplasma* infection in ticks, livestock and human in Ghaemshahr, Mazandaran province, Iran. J Arthropod Borne Dis. 2014; 8(2):204–211. 26114134 PMC4478432

[38] Swanson JS, Neitzel D, Reed KD, Belongia EA. Coinfections acquired from Ixodes ticks. Clin Microbiol Rev. 2006; 19:708–27. 10.1128/CMR.00011-06 17041141 PMC1592693

[39] Dunn JM, Krause PJ, Davis S, Vannier EG, Fitzpatrick MC, Rollend L, et al *Borrelia burgdorferi* promotes the establishment of *Babesia microti* in the Northeastern United States. PLoS One. 2014; 9:e115494 10.1371/journal.pone.0115494 25545393 PMC4278703

[40] Phillips RS, Wakelin D. *Trichuris muris*: effect of concurrent infections with rodent piroplasms on immune expulsion from mice. Exp Parasitol. 1976; 39:95–100. 1253888 10.1016/0014-4894(76)90015-1

[41] Persing DH. The cold zone: a curious convergence of tick-transmitted diseases. Clin Infect Dis. 1997; 25:S35–S42. 10.1086/516170 9233662

[42] Zakkyeh T, Mohammad Ali O, Nasibeh HV, Mohammad Reza YE, Farhang B, Fatemeh M. First molecular detection of *Theileria* ovis in *Rhipicephalus* sanguineus tick in Iran. Asian Pac J Trop Med. 2012; 5(1):29–32. 10.1016/S1995-7645(11)60240-X 22182639

