# Supplementary figures and images for: Epidemiology, risk factors, and co-infection of vector-borne pathogens in goats from Sistan and Baluchestan province, Iran
Source: PLoS One. 2019 Jun 20;14(6):e0218609. doi: 10.1371/journal.pone.0218609 (PMC6586321; doi:10.1371/journal.pone.0218609)

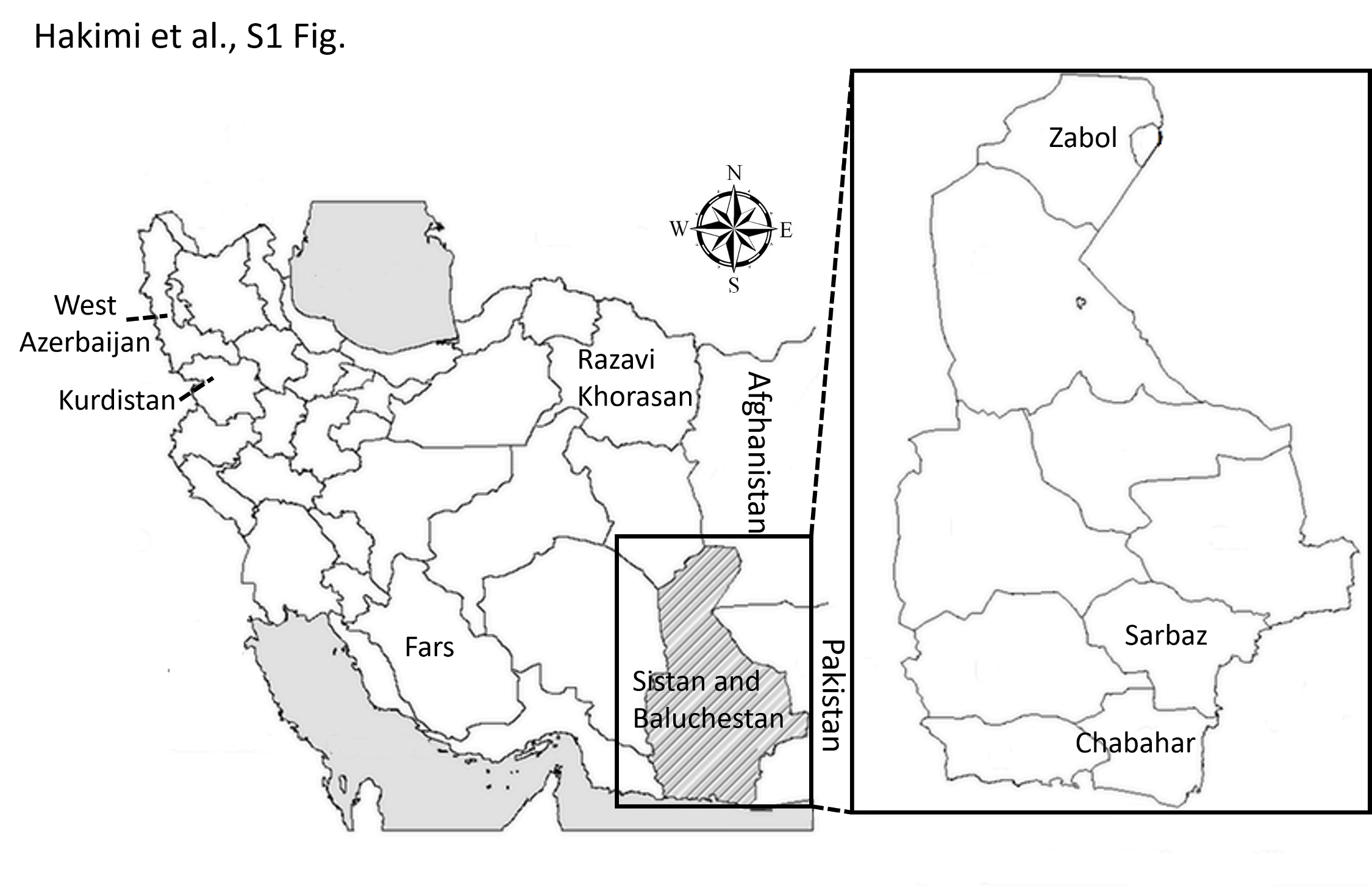

Supplement: S1 Fig — (TIF) [file pone.0218609.s001.tif]
